# Effectiveness of Digital Health Interventions for Chronic Obstructive Pulmonary Disease: Systematic Review and Meta-Analysis

**DOI:** 10.2196/76323

**Published:** 2025-05-26

**Authors:** Miaoqing Zhuang, Intan Idiana Hassan, Wan Muhamad Amir W Ahmad, Azidah Abdul Kadir, Xiaodong Liu, Furong Li, Yinuo Gao, Yang Guan, Shuting Song

**Affiliations:** 1 Department of Nursing College of Nursing and Rehabilitation Xi'an Jiaotong University City College Xi 'an China; 2 School of Health Sciences Health Campus Universiti Sains Malaysia Kota Bharu Malaysia; 3 School of Dental Sciences, Health Campus, Universiti Sains Malaysia Kelantan Malaysia; 4 School of Electronic Science and Technology University of Electronic Science and Technology of China Chengdu China; 5 Pneumology Department Shaanxi Provincial Hospital of Traditional Chinese Medicine Xi'an China

**Keywords:** mobile health, mHealth, telemedicine, self-management, remote patient monitoring, chronic respiratory disease, evidence synthesis

## Abstract

**Background:**

Chronic obstructive pulmonary disease (COPD), marked by dyspnea, cough, and sputum production, significantly impairs patients’ quality of life and functionality. Effective management strategies, particularly those empowering patients to manage their condition, are essential to reduce this burden and health care use. Digital health interventions—such as mobile apps for symptom tracking, wearable sensors for vital sign monitoring, and web-based pulmonary rehabilitation programs—can enhance self-efficacy and promote greater patient engagement. By improving self-management skills, these interventions also help alleviate pressure on health care systems.

**Objective:**

This systematic review and meta-analysis assesses the clinical effectiveness of smartphone apps, wearable monitors, and web-delivered platforms in four COPD management areas: (1) quality of life (measured by the COPD Assessment Test [CAT] and St George’s Respiratory Questionnaire), (2) self-efficacy (assessed by the General Self-Efficacy Scale), (3) functional capacity (evaluated via the 6-minute walk test and Modified Medical Research Council Dyspnea Scale), and (4) health care use (indicated by hospital and emergency department visits).

**Methods:**

A systematic review was conducted using predefined search terms in PubMed, Embase, Cochrane, and Web of Science up to January 26, 2025, to identify randomized trials on digital health interventions for COPD. Two reviewers independently screened studies and extracted data. Outcomes included quality of life, self-efficacy, functional status, and health care use.

**Results:**

This review included 17 studies with 2027 participants from 11 countries. Eleven trials involved health care professionals in digital platform use, and 12 reported adherence strategies. Digital tools for COPD primarily focused on telerehabilitation (eg, video-guided exercises) and self-management systems (eg, artificial intelligence–driven exacerbation alerts). The study participants were predominantly older adults. Meta-analysis results indicated that digital health interventions significantly improved quality of life at 3 months on the CAT (mean difference [MD] −1.65, 95% CI –3.17 to –0.14; *P*=.03); at 6 months on the CAT (MD −2.43, 95% CI −3.93 to −0.94; *P*=.001) and St George’s Respiratory Questionnaire (MD 3.25, 95% CI 0.69-5.81; *P*=.01); at 12 months on the CAT (MD −2.53, 95% CI −3.91 to −1.16; *P*<.001), EQ-5D (MD 0.04, 95% CI 0.01-0.07; *P*=.02), and EQ-5D visual analogue scale (MD 5.88, 95% CI 0.38-11.37; *P*=.04); the General Self-Efficacy Scale at 3 months (MD 1.65, 95% CI 0.62-2.69; *P*=.002) and 6 months (MD 1.94, 95% CI 0.83-3.05; *P*<.001); and the Modified Medical Research Council Dyspnea Scale at more than 3 months (MD −0.23, 95% CI −0.36 to −0.11; *P*=.003). However, no significant differences were observed in the 6-minute walk test, emergency department admissions, hospital admissions, emergency department admissions for COPD, or hospital admissions for COPD.

**Conclusions:**

Our findings suggest that digital health interventions may benefit COPD patients, but their clinical effectiveness remains uncertain. Further robust studies are needed, particularly those involving larger numbers of older adults with COPD.

**Trial Registration:**

PROSPERO CRD420251032053; https://www.crd.york.ac.uk/PROSPERO/view/CRD420251032053

## Introduction

### Background

Chronic obstructive pulmonary disease (COPD), the third leading global cause of mortality, imposes a disproportionate burden on low- and middle-income countries, where >90% of its 384 million cases occur [1,2]. In China alone, COPD affects 13.7% of adults aged ≥40 years, driving substantial disability and health care expenditures that threaten household economic stability [3,4]. While current guidelines emphasize multidisciplinary care, systemic barriers such as workforce shortages and fragmented care pathways limit implementation, particularly in resource-constrained settings [5].

The growing imperative for scalable, patient-centered solutions has catalyzed interest in digital health technologies. Telehealth platforms, wearable sensors, and artificial intelligence (AI)–driven predictive tools now enable remote symptom monitoring, personalized rehabilitation, and real-time clinician-patient communication, potentially overcoming geographical and financial access barriers [6,7]. Accelerated by the COVID-19 pandemic, these innovations align with the World Health Organization’s priorities for integrating digital tools into chronic disease frameworks [8].

Despite proliferating trials evaluating COPD-focused digital interventions ranging from smartphone-based pulmonary rehabilitation to smart inhalers with adherence tracking, critical evidence gaps persist. Earlier reviews either examined narrow subsets (eg, telemonitoring alone) or aggregated heterogeneous technologies (eg, combining SMS text message reminders with AI systems), obscuring the efficacy of advanced tools such as sensor-guided activity adapters or exacerbation prediction algorithms [9-12]. No synthesis has specifically assessed whether contemporary digital health tools (1) improve COPD-specific quality of life (QoL) and self-efficacy, (2) enhance functional capacity, or (3) reduce acute care use—knowledge essential for guiding clinical adoption.

**Figure 1:**
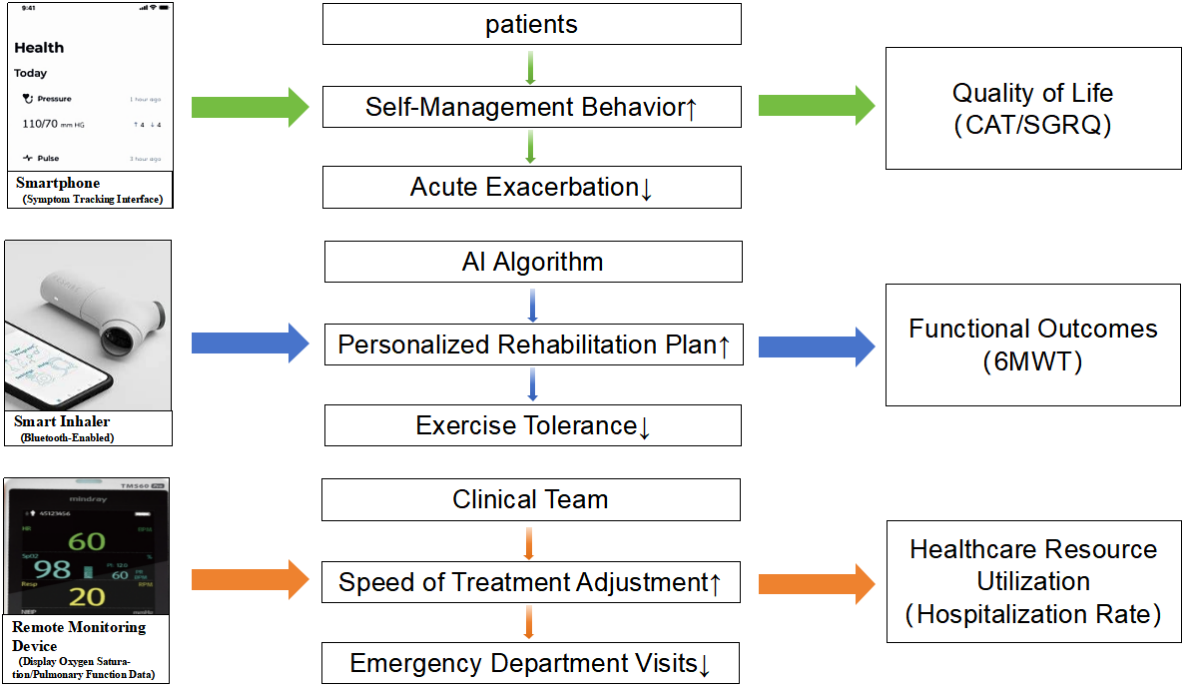
Hypothesized pathways.

The hypothesized pathways through which digital health tools may influence COPD outcomes are illustrated in Figure 1. We propose that these interventions operate via three synergistic mechanisms: (1) enhanced self-management via mobile apps and wearable sensors, which provide real-time feedback on symptoms (eg, dyspnea thresholds) and medication adherence, enabling early detection of exacerbations, possibly reducing hospitalization risk by prompting preemptive interventions [13]; (2) personalized, AI-driven telerehabilitation platforms that adapt exercise regimens based on continuous spirometry or oxygen saturation data, potentially improving functional capacity and QoL through optimized physical activity [14]; and (3) clinician-patient synergy and remote monitoring systems to facilitate data sharing between patients and care teams, allowing timely adjustment of treatment plans (eg, corticosteroid titration during exacerbations), which could decrease emergency visits [15].

This framework posits that effective tools must concurrently engage patients (via behavioral nudges) and clinicians (through actionable analytics) to disrupt the cycle of COPD deterioration. This systematic review and meta-analysis addresses these gaps by evaluating rigorously designed digital health interventions targeting COPD management. By synthesizing evidence on patient-centered outcomes and health care use metrics, we aim to clarify the clinical value of these technologies and inform equitable implementation strategies.

### Objective

This systematic review and meta-analysis aims to assess whether current digital health tools for COPD management can (1) improve disease-specific QoL and self-efficacy, (2) enhance functional capacity, and (3) reduce the use of acute health care services—key outcomes necessary to guide clinical adoption and policy decisions.

## Methods

This systematic review and meta-analysis was conducted in compliance with the Preferred Reporting Items for Systematic Reviews and Meta-Analyses (PRISMA) guidelines [16], and the PRISMA 2020 Checklist was used (Multimedia Appendix 1). The protocol for this study was registered in the International Prospective Register of Systematic Reviews (CRD420251032053).

### Search Strategy

A systematic search of the PubMed, Embase, Cochrane, Web of Science, and Scopus databases was conducted up to January 26, 2025, to identify relevant publications. The search used combinations of keywords and indexing terms, such as MeSH (Medical Subject Headings) or Emtree, associated with the search domains. An automated electronic search was carried out using the MeSH terms identified in PubMed. The following MeSH keywords were included: “Pulmonary Disease, Chronic Obstructive” OR “chronic obstructive pulmonary disease” OR “COPD” OR “chronic obstructive lung disease” OR “chronic airflow obstruction” OR “emphysema” AND “digital health” OR “telehealth” OR “mHealth” OR “eHealth” OR “biosensor” OR “remote monitoring” OR “Smartphone” OR “Mobile Applications” OR “Apps” OR “Internet-based interventions” OR “Web-based platforms” AND “self-management” OR “self-monitoring” OR “self-care” AND “randomized controlled trial” OR “controlled clinical trial” OR “Clinical Trial.” A comprehensive search strategy for every database is outlined in Multimedia Appendix 2. Boolean operators were used to merge and cross-reference across different domains. Furthermore, a manual search was conducted by examining the reference lists of reviews on related topics and selected articles.

### Eligibility Criteria

The specific eligibility requirements of inclusion criteria used the population, intervention, comparison, outcomes, and study format: (1) the population was COPD; (2) the intervention was an intervention delivered via a web-based remote digital health management system; (3) the comparison was the control group that received only the usual care interventions and the intervention group that used the web-based remote digital health management system in addition to the usual care interventions; (4) the outcomes were the impacts of the interventions on overall or at least one type of relevant health-related outcomes (eg, QoL, self-efficacy, functional outcomes, and health care use indicators); and (5) the study design was randomized controlled trials (RCTs). As the inclusion of unpublished studies can potentially introduce bias [17], this systematic review and meta-analysis only considered publications from peer-reviewed journals.

The review excluded case reports; preprint papers; letters to editors; simulation studies; published conference abstracts; research lacking adequate details on the measurement of the outcomes of interest; qualitative studies; surveys or reviews; studies describing protocols, along with those focusing solely on the interface or internal structure of apps; and studies based on web pages or websites without associated applications.

### Study Selection and Data Extraction

Two researchers separately reviewed the identified papers to reduce potential errors and bias throughout the selection process. Initially, the authors screened the title and abstracts of the potential papers against the set inclusion and exclusion criteria. Following this, the final selection of papers was made after thoroughly reading the complete manuscripts of the qualifying papers and their references. Any discrepancies were settled through discussions among the authors until a consensus was reached.

A structured data extraction form was used to gather the following details: the first author’s name; year of publication; study type; participant demographics (age range, sex ratio, sample size, and country); interventions for remote digital health management; intervention duration; involvement of health care professionals (HCPs); strategies implemented to ensure participant compliance; and outcomes. The corresponding authors were contacted to clarify or obtain any information that was unclear or missing.

### Study Quality Evaluation

Two researchers performed the quality assessment independently. Any disagreements were resolved through discussion between the two researchers, and a third investigator independently reviewed the final decisions. The quality of the RCTs was evaluated by 2 researchers using the Cochrane Collaboration’s tool for assessing risk of bias [18]. This tool addresses 6 domains of bias: selection bias, performance bias, detection bias, attrition bias, reporting bias, and other biases. The risk of bias was classified as high, low, or unclear, with reasons provided for each classification.

### Statistical Analysis

All meta-analyses were conducted using RevMan (version 5.4; Cochrane Collaboration). The statistical indicators used included the standardized mean difference (SMD), odds ratio, and 95% CI. An inverse-variance-weighted linear meta-analysis of SMD (Hedges *g*) was carried out to evaluate the effect size of smartphone-based remote digital health management on changes in review outcomes, such as self-efficacy/Modified Medical Research Council (mMRC)/6-minute walk test (6MWT). In general, a Hedges *g* value of less than 0.2 suggests a small effect, approximately 0.5 indicates a moderate effect, and greater than 0.8 signifies a strong effect. Heterogeneity among the results was assessed using the *I*^2^ statistical test. The choice between a random-effects model and a fixed-effects model was based on the heterogeneity *I*^2^ test, with *I*^2^ values below 50% indicating the use of a fixed-effects model and values above 50% suggesting the use of a random-effects model [18]. Effect sizes were compared using *z* tests, and a *P* value <.05 was considered statistically significant.

## Results

### Study Selection

**Figure 2:**
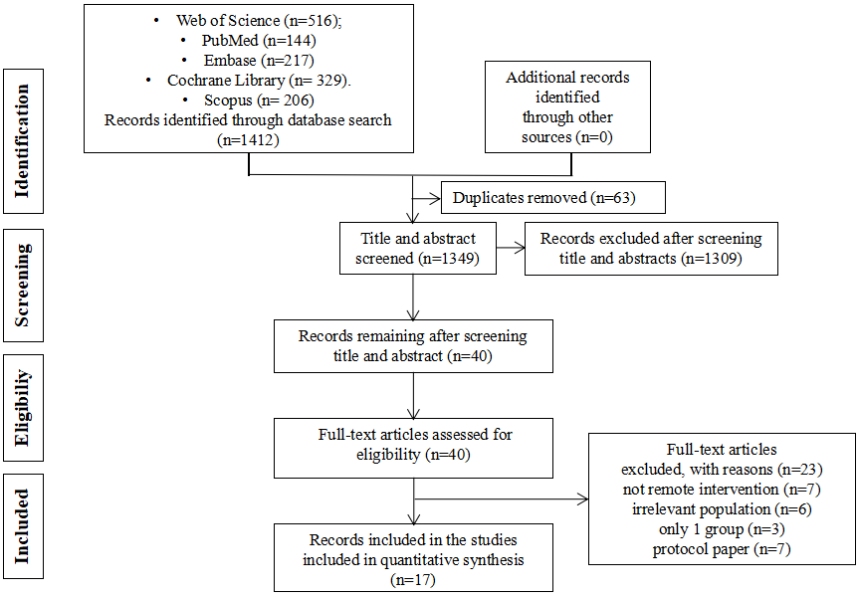
The PRISMA (Preferred Reporting Items for Systematic Reviews and Meta-Analysis) flow diagram.

The PRISMA flow diagram (Figure 2) illustrates the screening process and the criteria used for excluding papers. The initial search yielded 1206 citations, from which 63 duplicates were eliminated. Following the removal of duplicates, 1143 records were evaluated against the inclusion and exclusion criteria. Subsequently, 40 full-text manuscripts were examined for eligibility. Out of these 45 articles, 23 studies were excluded for various reasons. As a result, 17 records were ultimately included in this review.

### Quality of Studies

**Figure 3:**
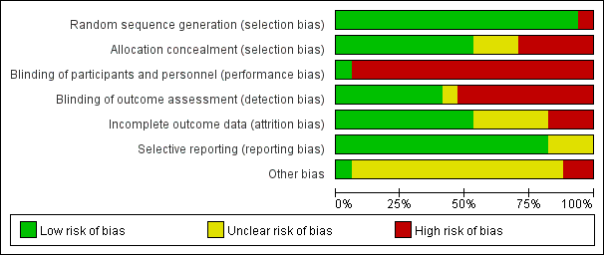
Risk of bias graph of randomized controlled trials.

**Figure 4:**
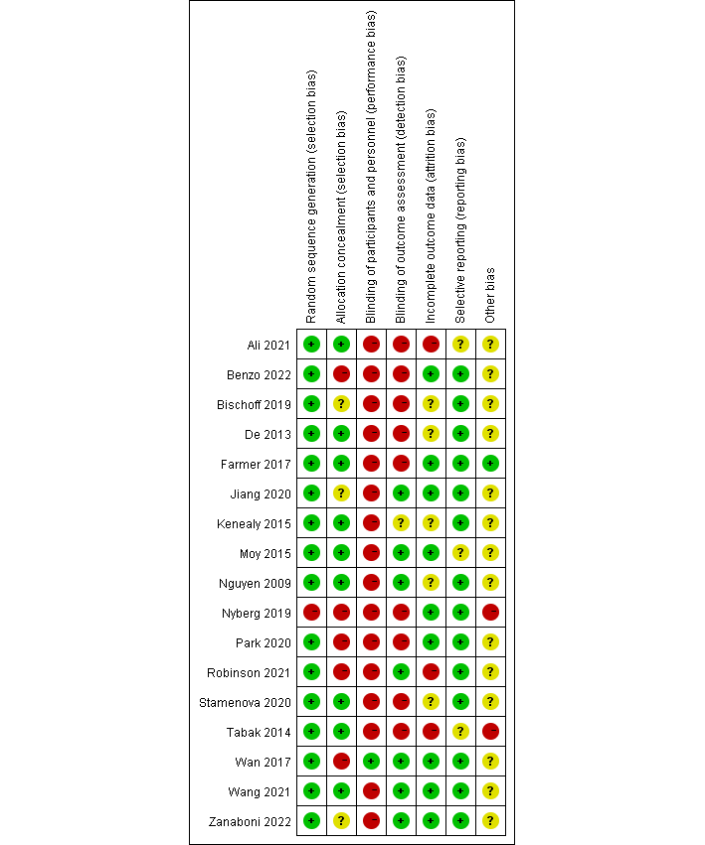
Risk of bias summary of randomized controlled trials. The review authors’ judgments about each risk of bias item are presented as percentages. The x-axis represents the percentage of studies that were found to have low (green), unclear (yellow), or high (red) risk of bias for each domain.

Figures 3 and 4 [17,19-34] show the risk of bias judgment of the RCTs. All the studies [17,19-34] used specific random sequence generation methods. Allocation concealment was rated as having a low risk of bias in 9 (53%) trials [21,22,24-26,29-32] and unclear bias in 4 (23.5%) trials [17,19,20,23]. Given the nature of web-based remote digital health management interventions, blinding of researchers and participants is unreasonable, which inevitably leads to risk deviation. In total, only 9 trials [17,19-22,27,28,31,33] had a low risk of incomplete outcome data; it is worth noting that 14 trials [17,19-28,32-34] had a low risk of selective outcome reporting. The rates of dropout and attrition were within acceptable limits.

### Characteristics of the Included Studies

Table 1 provides a summary of the specific information extracted from the included studies.

**Table 1:** Specific information on the studies included.

Studies	Countries	Study design	Study type	Participants, n	Age (y), mean (SD)	Intervention duration (months), n	Intervention and control arm	Involvement of health care professionals	Measures to ensure intervention adherence	Outcomes
Jiang et al [19], 2020	China	Clinical outcome	RCT^a^	E^b^: 53; C^c^: 53	E: 70.92(6.39); C: 71.83 (7.60)	6	Intervention: lung rehabilitation intervention plan based on WeChat; control: routine outpatient rehabilitation	Respiratory experts, clinicians, rehabilitation practitioners, nurses	Regularly calls	CAT^d^; mMRC^e^; SGRQ^f^
Park et al [20], 2020	Korea	Clinical outcome	RCT	E: 22; C: 20	E: 70.45 (9.40); C: 65.06 (11.12)	6	Intervention: smartphone-based self-management plan Control: telephone	A pulmonary physician and a nurse researcher	Send SMS text message	6MWT^g^; self-efficacy
Zanaboni et al [17], 2016	Norway, Australia, and Denmark	Clinical outcome	RCT	E: 40; C: 40	E: 64.9 (7.1); C: 63.5 (8.0)	24	Intervention: telerehabilitation; control: usual care	Physiotherapist	Send SMS text message	6MWT, EQ-5D; mMRC, CAT; self-efficacy
Wang et al [21], 2021	China	Clinical outcome	RCT	E: 39; C: 39	E: 63.2 (7.5); C: 64.4 (7.0)	12	Intervention: plans based on mobile health apps; control: usual care	A respiratory nurse	Not stated	CAT; self-efficacy
Farmer et al [22], 2017	England	Clinical outcome	RCT	E: 110; C: 56	E: 69.8 (9.1); C: 69.8 (10.6)	12	Intervention: digital health system; control: usual care	Doctor, nurse or physiotherapist	Postal reminders; telephone contact	SGRQ; EQ-5D
Boer et al [23], 2019	Netherlands	Clinical outcome	RCT	E: 35; C: 41	E: 69.3 (8.8); C: 65.9 (8.9)	12	Intervention: smart mobile health program; control: paper action plan	Not stated	Regularly calls	EQ-5D; EQ-5D visual analogue scale
Stamenova et al [24], 2020	Canada	Clinical outcome	RCT	E: 41; C: 20	E: 71.98 (9.52); C: 72.78 (9.16)	6	Intervention: remote monitoring; control: routine	In-person follow-up appointments and access to a certified respiratory educator; a respiratory therapist	Regularly calls	Emergency department admissions or for COPD^h^; hospital admissions or for COPD
De San Miguel et al [25], 2013	Australian	Clinical outcome	RCT	E: 36; C: 35	E: 71; C: 74	6	Intervention: remote monitoring; control: usual care	Not stated	Regularly calls	Emergency department admissions or for COPD; hospital admissions or for COPD
Kenealy et al [26], 2015	New Zealand	Clinical outcome	RCT	E: 98; C: 73	E: 62-83; C: 60-77	6	Intervention: telemedicine; control: usual care	3 respiratory physicians, 2 respiratory nurse specialists, and 1 respiratory nurse practitioner	Not stated	Self-efficacy; SGRQ
Nyberg et al [27], 2019	Sweden	pilot study	RCT	E: 43; C: 40	E: 65 (7); C: 71 (8)	12	Intervention: remote self-management; control: usual care	4 asthma or COPD nurses, 1 district nurse, 1 dietician, and 1 physiotherapist	Not stated	CAT; mMRC; EQ-5D
Benzo et al [28], 2022	United States	Clinical outcome	RCT	E: 188; C: 187	E: 69.335 (9.53); C: 68.676 (9.53)	3	Intervention: remote monitoring; control: usual care	Not stated	Regularly calls	mMRC
Tabak et al [29], 2014	Netherlands	Pilot study	RCT	E: 12; C: 12	E: 64.1 (9.0); C: 62.8 (7.4)	3	Intervention: telemedicine program; control: usual care	A chest physician or nurse practitioner	Regularly calls	6MWT; EQ-5D; EQ-5D visual analogue scale
Ali et al [30], 2021	Sweden	Clinical outcome	RCT	E: 110; C: 112	E: 71.1 (9.8); C: 70.4 (9.1)	6	Intervention: digital-based intervention; control: usual care	Not stated	Regularly calls	Self-efficacy
Moy et al [31], 2015	United States	Clinical outcome	RCT	E: 144; C: 77	E: 67.0 (8.6); C: 66.4 (9.2)	3	Intervention: internet-mediated pedometer-based intervention; control: usual care	Not stated	Not stated	SGRQ
Nguyen et al [32], 2009	United States	Pilot study	RCT	E: 8; C: 9	E: 64.0 (12); C: 72 (9)	6	Intervention: mobile phone –based rehabilitation; control: routine rehabilitation	Nurses	Send SMS text message	SGRQ
Wan et al [33], 2017	United States	Clinical outcome	RCT	E: 57; C: 52	E: 68.4 (8.7); C: 66.8 (7.9)	3	Intervention: web-based pedometer intervention; control: individual pedometer intervention	Not stated	Not stated	6MWT; SGRQ; mMRC
Robinson et al [34], 2021	United States	Clinical outcome	RCT	E: 93; C: 92	E: 69.2 (7.2); C: 70.4 (7.3)	6	Intervention: web-based exercise routine intervention; control: usual care	Not stated	Follow-up, in-person assessments	6MWT; SGRQ; mMRC

^a^RCT: randomized controlled trial.

^b^E: experimental group.

^c^C: control group.

^d^CAT: Chronic Obstructive Pulmonary Disease Assessment Test.

^e^mMRC: Modified Medical Research Council.

^f^SGRQ: St George’s Respiratory Questionnaire.

^g^6MWT: 6-minute walk test.

^h^COPD: chronic obstructive pulmonary disease.

A total of 17 studies were published between 2009 and 2023. All studies compared either the use of a single app/web platform or an app/web platform combined with additional participant support against a control group. Collectively, these studies involved 2027 participants, with sample sizes ranging from 17 [32] to 375 [28]. Geographically, 5 studies [28,31-34] were conducted in the United States; 2 studies in China [19,21], Australian [17,25], the Netherlands [23,29], and Sweden [27,30]; and 1 study in Korea [20], Norway [17], Denmark [17], England [22], Canada [24], and New Zealand [26]. In terms of study design, all the studies were RCTs. A total of 14 articles [17,19-26,28,30,31,33,34] reported clinical outcomes, whereas 3 [27,29,32] were pilot studies. Regarding the duration of interventions, 4 studies lasted ≤3 months [11,12,14,16], 9 studies [19,20,24-27,30,32,34] lasted between 3 and 6 months, 3 studies [17,21,27] lasted between 6 and 12 months, and 3 studies [17,22,23] lasted ≥12 months. All of the participants in these studies were aged ≥60 years.

In 15 trials, the control group received standard care without the use of the app [17,19-23,25-31,33,34]. In 1 trial, they received routine in-person follow-up appointments and access to a certified respiratory educator [24] or as per the physician’s judgment based on current guidelines [30], while in 4 trials, they were provided with leaflets [22], paper action plan [23], a COPD book [25], or an educational packet of 12 self-management themes [28]. Telephone follow-up was used in 2 trials [20,26], and in 1 trial, the control group used the same app as the intervention group but with different functionality [32].

### Involvement of HCPs and Strategies to Ensure Adherence via Digital Health Tool Management Interventions

A total of 11 trials [17,19-22,24,26,27,29,30,32] included the participation of HCPs in digital health tools use, while the remaining 6 trials [23,25,28,31,33,34] did not provide specific information regarding HCP involvement in digital health tools use (Table 1). The roles of HCPs varied across the trials; in most cases, HCPs were involved in prescribing rehabilitation or self-management training for patients [19,20,22,27,29,30,32], guiding the training process [17,29], installing the digital health equipment and instructing patients on how to use them [21,22,25,26,29,30], and reviewing the data [17,22,24,26]. A total of 3 trials described approaches to enhance adherence to the digital health tools management interventions. The strategies involved checking progress [29], scheduling the follow-up meetings [30], sending regular SMS text message reminders, and making periodic phone calls [32].

### Digital Health Tools’ Characteristics

Across the studies, a total of 17 distinct digital health tools were used. These tools were deployed across various types of digital platforms, including 1 WeChat official account, 5 apps, 7 websites, and 4 that did not specify the platform’s details. The functionality of these platforms varied significantly across the trials. Within the context of COPD management, digital platforms were used in 2 key areas: self-management [20-31] and rehabilitation [17,19,32-34]. Concerning self-management, most digital health platforms feature functionalities such as self-monitoring, medication reminders, health information provision, assessment, feedback, access to health services, and social support. In the realm of rehabilitation, these digital health platforms are capable of tracking various physical activities and transmitting rehabilitation-related data to a server, allowing therapists to review this information. Notably, most of these tools require additional devices to fully realize their capabilities. Furthermore, certain tools facilitate patients’ access to visual and auditory feedback on their exercises by displaying real-time information on connected device screens. An overview of digital health tools characteristics is provided in Table 2.

**Table 2:** Digital health tools characteristics.

Studies	Digital health tools name	General objective	Specific objectives	Devices	Main features of the digital health tools
Jiang et al [19], 2020	PeR	Rehabilitation	Improve quality of life, symptoms, and exercise self-efficacy	WeChat official account	PeR management on the basis of evaluation, including respiratory training, sports training, diet guidance, and medication knowledge
Park et al [20], 2020	SASMP	Self-management	Self-care behavior	An Android platform (version 2.3; Gingerbread)	Education, exercises, self-monitoring of symptoms and exercise, and social support
Zanaboni et al [17], 2016	Not stated	Rehabilitation	Reducing hospital readmissions	A treadmill, a pulse oximeter, a tablet computer, and a holder for the tablet computer	An integrated intervention consisting of exercise training at home, telemonitoring, and self-management Access the individual training program, fill in a daily diary and a training diary, review historical data, exchange electronic messages, schedule videoconferencing sessions, and facilitate individual goal setting and goal attainment
Wang et al [21], 2021	Not stated	Self-management	Quality of life, self-management behavior and exercise and smoking cessation behavior	No device	Provided knowledge and information support to participants. Provided visual aids to teach participants skills to manage the disease. Provided motivational support to participants, which included a peer support chat room and an expert support portal
Farmer et al [22], 2017	EDGE	Self-management	Improve quality of life and clinical outcomes	Android tablet computer (Samsung Galaxy Tab) running the app software and a Bluetooth-enabled oximeter probe	Provide monitoring and self-management support Complete the symptom diary and recorded their oxygen saturation and heart rate Set alerts when the vital sign value goes above (or below)
Boer et al [23], 2019	Not stated	Self-management	The number of exacerbation-free weeks	A mobile phone, a pulse oximeter (CMS50D, Contec Medical Systems), a spirometer (PiKo-1 monitor, nSpire), and a forehead thermometer (FTN, Medisana AG)	Provide predictions of early exacerbation onset and timely treatment advice without the interference of health care professionals
Stamenova et al [24], 2020	Not stated	Self-management	Self-management	A custom tablet computer, a Pulsewave wrist cuff monitor (which measures blood pressure), an oximeter, a weighing scale, and a thermometer	Record vital statistics on the Cloud DX platform. Provide a written version of a personalized COPD action plan
De San Miguel et al [25], 2013	Health HUB	Self-management	Reduce the incidence of hospitalizations and ED^a^ presentations	No device	Measure and record their vital signs (blood pressure, weight, temperature, pulse, and oxygen saturation levels) on a daily basis. Transmitted signs automatically via telephone to a secure website where they were monitored each day by the telehealth nurse
Kenealy et al [26], 2015	Health HUB	Self-management	Quality of life; self-care; hospital use; costs; and the experiences of patients, informal carers, and health care professionals	An LCD^b^	Enter the data into a commercially-available electronic device that uploaded data once a day to a nurse-led monitoring station peer support chat room and an expert support portal
Nyberg et al [27], 2019	COPD Web	Self-management	Increasing PA^c^	No device	The COPD Web includes, texts, pictures, and videos as well as interactive components such as a tool for registration of PA, including automated feedback
Benzo et al [28], 2022	Not stated	Self-management	Improves the physical and emotional disease-specific quality of life	A computer tablet, a Garmin Vivofit activity monitor and an oximeter (Nonin 3150)	Capture daily steps and self-report of symptoms. Send and receive messages to or from the coach. The data gathered on the tablet via Bluetooth (steps, compliance with daily exercises, messages, and symptoms) was transmitted to a server and, ultimately, to web-based patient portal
Tabak et al [29], 2014	Condition Coach	Self-management	The number of hospitalizations, length of stay, and ED visits	An accelerometer-based activity sensor (Inertia Technology, Enschede) and a smartphone (Desire S; HTC)	Activity coach for ambulant activity monitoring and real-time coaching of daily activity behavior. Web-based exercise program for home exercising. Self-management of COPD^d^ exacerbations via a triage diary on the web portal, including self-treatment of exacerbations. Teleconsultation
Ali et al [30], 2021	Not stated	Self-management	General self-efficacy and hospitalization or death	No device	PCC^e^ using a combined digital platform and structured telephone support system
Moy et al [31], 2015	Not stated	Self-management	Increasing PA	A computer with an internet connection; a USB port; and Windows XP, Vista, 7, or 8	A novel internet-mediated, pedometer-based exercise intervention
Nguyen et al [32], 2009	Not stated	Rehabilitation	Maximal workload, 6-minute walk distance, health-related quality of life, or total steps	An Omron HJ-112 digital pedometer	Develop an individualized exercise plan, were issued a pedometer and exercise booklet. Instructed to continue to log their daily exercise and symptoms. MOBILE-Coached also received weekly reinforcement text messages on their cell phones; reports of worsening symptoms were automatically flagged for follow-up
Wan et al [33], 2017	ESC	Rehabilitation	Increasing PA	An Omron HJ-720 ITC pedometer	A pedometer and website which provided goal setting, feedback, disease-specific education, and a web-based community forum Pedometer alone for 3 months
Robinson et al [34], 2021	Not stated	Rehabilitation	Increasing PA	Pedometer	Personalized, progressive step-count goals, feedback, web-based COPD-related education and social support via a web-based community

^a^ED: emergency department.

^b^LCD: liquid crystal display.

^c^PA: physical activity.

^d^COPD: chronic obstructive pulmonary disease.

^e^PCC: person-centered care.

### Intervention Effectiveness

#### QoL Outcomes

An array of QoL outcomes was measured across the trials, which included a COPD Assessment Test (CAT), St George’s Respiratory Questionnaire (SGRQ), EQ-5D and EQ-5D visual analogue scale (VAS). Assessments were conducted at 3 different time points: 3, 6, and 12 months.

**Figure 5:**
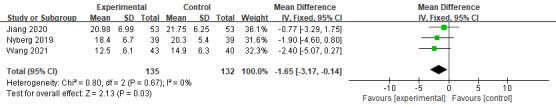
Meta-analysis results and forest plot of the digital health interventions on quality of life assessed by the Chronic Obstructive Pulmonary Disease Assessment Test at 3 months.

**Figure 6:**
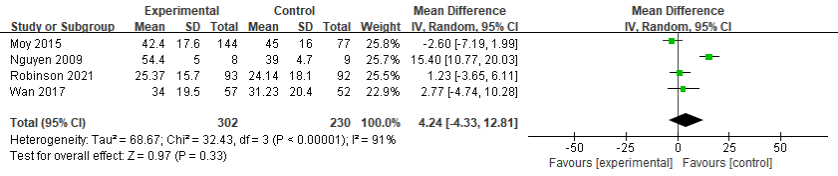
Meta-analysis results and forest plot of the digital health interventions on quality of life assessed by St George’s Respiratory Questionnaire at 3 months.

**Figure 7:**
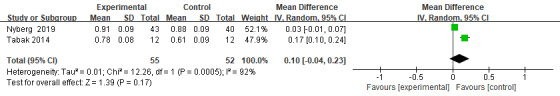
Meta-analysis results and forest plot of the digital health interventions on quality of life assessed by EuroQol 5 Dimensions at 3 months.

**Figure 8:**
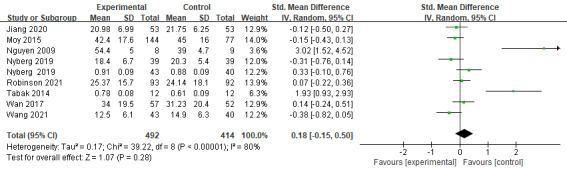
Meta-analysis results and forest plot of the digital health interventions on quality of life at 3 months.

At 3 months, the effects of digital health management were limited but showed some promising trends. A total of 3 studies (n=267) assessed its impact on CAT scores, revealing a mild but significant effect size (Hedges *g*=−1.65; mean difference [MD] −1.65, 95% CI −3.17 to −0.14; *P*=.03; Figure 5 [19,21,27]) with no heterogeneity (*I*^2^=0%). However, 4 studies (n=532) evaluating SGRQ scores found no significant effect (MD 4.24, 95% CI −4.33 to 12.81; *P*=.33; Figure 6 [31-34]) and high heterogeneity (*I*^2^=91%). Similarly, 2 studies (n=107) on EQ-5D also showed no significant improvement (MD 0.10, 95% CI −0.04 to 0.23; *P*=.17; Figure 7 [27,29]) with high heterogeneity (*I*^2^=92%). The overall meta-analysis (n=639) indicated that digital health management did not significantly improve QoL (SMD 0.18, 95% CI −0.15 to 0.50; *P*=.28; *I*^2^=80%; Figure 8 [19,21,27,29,31-34]).

**Figure 9:**
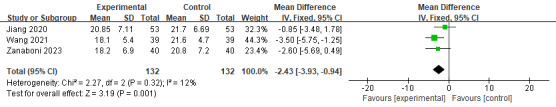
Meta-analysis results and forest plot of the digital health interventions on quality of life assessed by the Chronic Obstructive Pulmonary Disease Assessment Test at 6 months.

**Figure 10:**
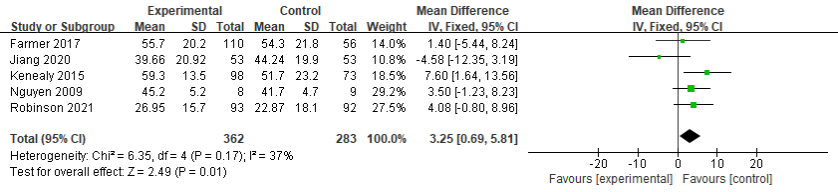
Meta-analysis results and forest plot of the digital health interventions on quality of life assessed by St George’s Respiratory Questionnaire at 6 months.

At 6 months, the benefits of digital health management became more pronounced. A total of 3 RCTs (n=264) examining CAT scores revealed a statistically significant benefit for the intervention group compared with controls (MD −2.43, 95% CI 3.93 to −0.94; *P*=.001; Figure 9 [17,19,21]), with low heterogeneity (*I*^2^=12%). In addition, 1 study on SGRQ scores also showed a significant effect (MD 3.25, 95% CI 0.69-5.81; *P*=.01; *I*^2^=37%; Figure 10 [19,22,26,32,34]), indicating improved symptom control and health status over time.

**Figure 11:**
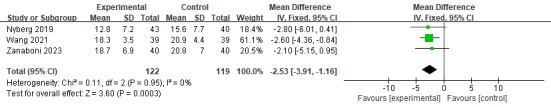
Meta-analysis results and forest plot of the digital health interventions on quality of life assessed by the Chronic Obstructive Pulmonary Disease Assessment Test at 12 months.

**Figure 12:**
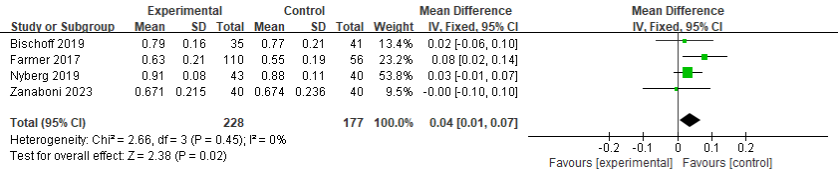
Meta-analysis results and forest plot of the digital health interventions on quality of life assessed by EQ-5D at 12 months.

**Figure 13:**
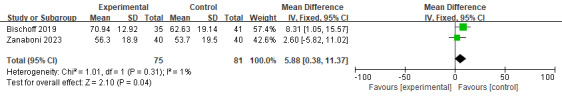
Meta-analysis results and forest plot of the digital health interventions on quality of life assessed by EQ-5D VAS at 12 months.

At 12 months, the positive effects observed at 6 months were sustained, with findings showing statistically significant improvements across multiple outcomes. A total of 5 studies (n=802) assessing CAT, EQ-5D, and EQ-5D VAS scores demonstrated highly significant effects in favor of digital health management (*P*＜.05), with no or low heterogeneity (Figure 11 [17,21,27], Figure 12 [17,22,23,27], and Figure 13 [17,23]). These results suggest that long-term intervention has a sustained impact on symptom relief and overall health status improvement.

#### Self-Efficacy

**Figure 14:**
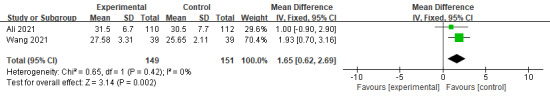
Meta-analysis results and forest plot of the digital health interventions on self-efficacy at 3 months.

**Figure 15:**
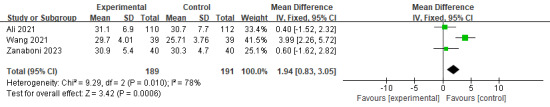
Meta-analysis results and forest plot of the digital health interventions on self-efficacy at 6 months.

**Figure 16:**
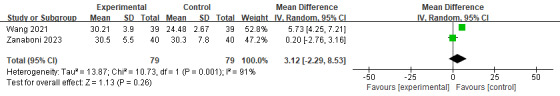
Meta-analysis results and forest plot of the digital health interventions on self-efficacy at 12 months.

Self-efficacy was also evaluated at 3, 6, and 12 months, using the General Self-Efficacy (GSE) Scale. Analysis showed significant differences at 3 months (MD 1.65, 95% CI 0.62-2.69; *P*=.002; Figure 14 [21,30]) and 6 months (MD 1.94, 95% CI 0.83-3.05; *P*<.001; Figure 15 [17,21,30]). Among the outcomes that were reported by 2 studies, no significant difference at 12 months (MD 3.12, 95% CI −2.29 to 8.53; *P*=.26) was observed between the two groups, with high statistical heterogeneity (*I*^2^=91%; Figure 16 [17,21]).

### Functional Outcomes

#### Dyspnea

**Figure 17:**
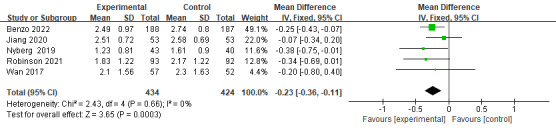
Meta-analysis results and forest plot of the digital health interventions on dyspnea at 3 months.

**Figure 18:**
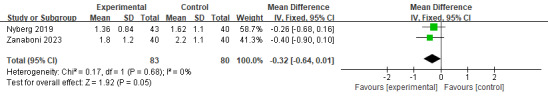
Meta-analysis results and forest plot of the digital health interventions on dyspnea at 12 months.

**Figure 19:**
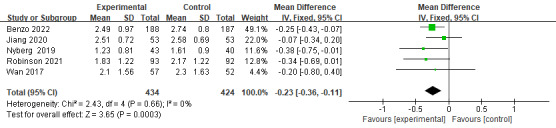
Meta-analysis results and forest plot of the digital health interventions on dyspnea at 6 months.

A total of 6 studies (with a total sample size of 1392 patients) assessed the effect of digital health management on the mMRC Dyspnea Scale. The meta-analysis showed a significant difference favoring digital health management at 3 months (MD −0.23, 95% CI −0.36 to −0.11; *P*<.001; *I*^2^=0%; Figure 17 [19,27,28,33,34]) and at 12 months (MD −0.32, 95% CI −0.64 to 0.01; *P*=.05; *I*^2^=0%; Figure 18 [17,27]). However, no significant difference was observed at 6 months, with moderate heterogeneity among studies (MD −0.17, 95% CI −0.46 to 0.12; *P*=.26; *I*^2^=50%; Figure 19 [19,27,28,33,34]).

#### Six-Minute Walk Test

**Figure 20:**
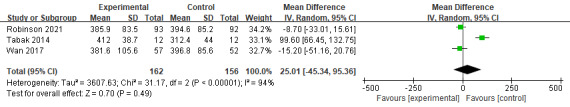
Meta-analysis results and forest plot of the digital health interventions on 6-Minute Walk Test at 3 months.

**Figure 21:**
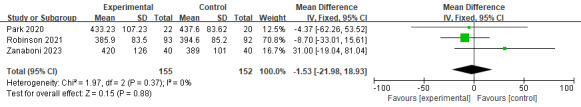
Meta-analysis results and forest plot of the digital health interventions on 6-Minute Walk Test at 6 months.

Meta-analysis indicated a positive trend for digital health management in 6MWT. However, no significant differences were observed between the intervention and control groups at 3 months (MD 25.01, 95% CI −45.34 to 95.36; *P*=.49; *I*^2^=94%; Figure 20 [29,33,34]) or at 6 months (MD −1.53, 95% CI −21.98 to 18.93; *P*=.88; *I*^2^=0%; Figure 21 [17,20,34]).

#### Health Care Use Indicators

**Figure 22:**
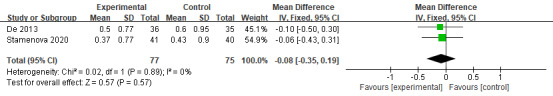
Meta-analysis results and forest plot of the digital health interventions on emergency department admissions.

**Figure 23:**
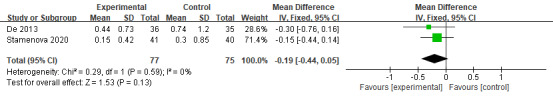
Meta-analysis results and forest plot of the digital health interventions on hospital admissions.

**Figure 24:**
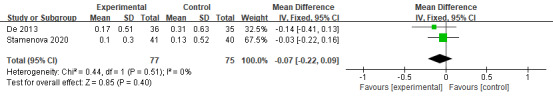
Meta-analysis results and forest plot of the digital health interventions on emergency department admissions for chronic obstructive pulmonary disease.

**Figure 25:**
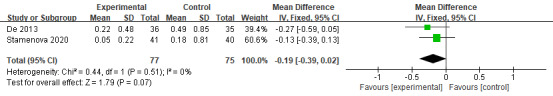
Meta-analysis results and forest plot of the digital health interventions on hospital admissions for chronic obstructive pulmonary disease.

Health care use indicators included emergency department admissions, hospital admissions, emergency department admissions for COPD and hospital admissions for COPD. The overall effect revealed that the digital health management intervention could not effectively reduce health care use indicators. The analysis showed no significant differences in emergency department admissions (MD −0.08, 95% CI −0.35 to 0.19; *P*=.57; Figure 22 [24,25]), hospital admissions (MD −0.19, 95% CI −0.44 to 0.05; *P*=.13; Figure 23 [24,25]), emergency department admissions for COPD (MD −0.07, 95% CI −0.22 to 0.09; *P*=.40; Figure 24 [24,25]), and hospital admissions for COPD (MD −0.19, 95% CI −0.39 to 0.02; *P*=.07; Figure 25 [24,25]), all of them with no statistical heterogeneity (*I*^2^=0%).

## Discussion

### Principal Findings

This systematic review and meta-analysis aggregated data from 17 RCTs, encompassing a total of 2027 participants, to assess the efficacy of digital health interventions aimed at COPD. The digital health tools were mainly used for rehabilitation and self-management purposes. Although many trials included HCPs in the intervention process, few elaborated on strategies to maintain participant adherence throughout the intervention period. Most participants were older adults. Our study results showed that the remote digital health intervention demonstrated significant effects in improving QoL. Specifically, the CAT score showed statistically significant improvements at 3, 6 (CAT and SGRQ), and 12 months (CAT and EQ-5D and its VAS). In addition, the degree of dyspnea (mMRC) significantly decreased at 3 months and approached significance at 12 months. Self-efficacy (GSE) also improved significantly in the short term, particularly at 3 and 6 months, but no significant changes were observed at 12 months. Despite these encouraging results, no significant improvements were observed in physical performance (eg, the 6MWT) or hospitalization rates. For instance, the 6MWT did not show significant effects at either 3 months or 6 months. Similarly, although there were trends toward reduced all-cause hospitalization rates and COPD-related hospitalization rates, these did not reach statistical significance. While these results are promising, they should be interpreted with caution due to differences in digital health tools characteristics, content variability, and the diverse methodologies and clinical settings across studies, alongside generally small sample sizes.

### Effects of Interventions on the Health Outcomes of Patients With COPD

#### QoL Outcomes

The overall effect indicated that the QoL of the group using digital health interventions was superior to that of the control group, with effects at 12 months being more pronounced than at 3 and 6 months. This sustained improvement can be attributed to the benefits of prolonged treatment without requiring a therapist’s online presence, ensuring offline monitoring of rehabilitative activities [35]. This finding aligns with the technology acceptance model [36], where prolonged exposure enhances perceived usefulness (eg, recognizing the role of tools in exacerbation prevention). However, early dropout may reflect low perceived ease of use among older users struggling with multidevice complexity. Therefore, while sustained engagement leads to better health outcomes, addressing usability challenges is crucial, especially for older adults [37].

The improvements in CAT, SGRQ, EQ-5D, and EQ-5D VAS scores hold clinical significance, particularly when considering their impact on patient outcomes. Taking CAT as an example, the improvement (an average reduction of 1.65 points), although slightly below the minimal clinically important difference (2 points), may indicate mild symptom relief, such as reductions in dyspnea and coughing. This can have practical importance for daily functioning, especially for patients with a higher symptom burden. While these improvements do not directly translate into significant enhancements in overall QoL, they may help reduce the risk of acute exacerbations or slow disease progression, serving as a valuable supplementary tool for personalized management, particularly in resource-limited or remote health care settings.

Notably, while the 12-month QoL data suggest potential long-term benefits, the effect sizes are derived from only 5 studies with varying designs and high heterogeneity (*I*^2^ up to 92%). Due to the limitations of the available data, these long-term findings should be interpreted with caution. In addition, we emphasize the need for further high-quality, long-term studies to confirm the robustness and generalizability of these results.

#### Functional Outcomes

In the second subgroup analysis, digital health interventions showed the greatest impact on dyspnea in patients with COPD at 3 months, likely due to high adaptability and initial learning effects. However, no significant differences were observed at 6 months, suggesting a decline in novelty and short-term adjustable factors reaching their limit. A marginally significant improvement was noted at 12 months, indicating that long-term adherence may gradually alleviate chronic issues. Individual differences, environmental changes, and study design characteristics also contribute to these observations. These findings highlight the dynamic and complex nature of digital health interventions. Future longitudinal studies should include larger, more diverse patient populations and conduct detailed analyses of intervention effects over time to validate these results and explore underlying mechanisms.

Despite significant improvements in dyspnea (mMRC) and QoL (CAT/SGRQ) for patients with COPD through digital health interventions, no such benefits were seen in the 6MWT. This may be due to a mismatch between interventions and functional improvement needs. Most digital tools focused on symptom monitoring (eg, dyspnea diaries) and self-management education (eg, medication reminders), lacking structured exercise prescriptions. Evidence shows that improving 6MWT requires high-intensity aerobic training, which remote monitoring alone cannot provide [38]. Only 1 trial included sensor-guided walking training; others did not prioritize exercise adherence [29]. In addition, the sensitivity of 6MWT is limited by comorbidities and testing conditions, diluting intervention effects. Future research should use accelerometers for more precise functional assessment. Furthermore, declining patient engagement over time, possibly due to “digital health fatigue,” where interest in repetitive tasks wanes, may reduce effectiveness [39]. No improvements in 6MWT or mMRC at 6 months suggest short-term effects diminish with reduced participation.

#### Self-Efficacy

Subgroup analysis results indicate that at 6 months, digital health interventions significantly enhanced self-efficacy in patients with COPD, but no significant effect was observed at 12 months. This pattern aligns with the social cognitive theory by Bandura [40]: early improvements (at 3 and 6 months) stem from mastery experiences (eg, symptom control via real-time feedback) and clinician-mediated social persuasion. Self-efficacy (GSE) improved significantly in the short term but showed no significant changes at 12 months. The lack of sustained adaptive reinforcement likely contributed to “digital health fatigue,” leading to attrition at 12 months [39]. Patients may have felt they maximized benefits within a 6-month period, experiencing the greatest benefit during this phase. This contrasts with long-duration trials where improvements plateaued. These findings also align with the study by Pierz et al [41], who noted digital health technologies enhance self-efficacy through remote management, improved communication, and personalized support. The improvement in self-efficacy indicates an increased confidence among patients in managing their disease, which may promote behavioral changes, such as better medication adherence and greater participation in physical activity.

#### Health Care Use Indicators

The reason for the lack of improvement in health care resource use is, first, that the monitoring systems in the 2 included studies lacked closed-loop response mechanisms—personalized plans by Stamenova et al [24] did not integrate automated clinical pathways, while nurse-led monitoring by De San Miguel et al [25] experienced response delays, preventing data from being promptly translated into preventive interventions. Second, there was insufficient patient-side execution efficacy: the complexity of multidevice operation in the former affected adherence among older patients, while the latter relied on self-recording, which was prone to errors. In addition, the interventions were not deeply integrated with regional health care resources (eg, emergency medication delivery and rapid referrals), meaning that even when deterioration was detected, timely treatment could not be ensured. Moreover, the technical design did not align with COPD management needs—the interfaces were complex and lacked threshold alerts for deterioration, resulting in inefficient data use. Fundamentally, the application of technology remained at the data collection level, failing to address systemic bottlenecks, such as the “monitoring-to-action” transition and resource accessibility, which explains why hospitalization rates were not reduced [24,25].

#### Analysis of High Heterogeneity

##### SGRQ Scores of QoL

The high heterogeneity in SGRQ scores (*I*^2^=91%) is primarily due to the multidimensional nature of the scale (symptoms, activity, and disease impact) conflicting with the varied focus of interventions. For instance, the study by Farmer et al [22] improved symptoms through blood oxygen monitoring but had limited effects on activity, while the study by Zanaboni et al [17] demonstrated greater improvements in activity via exercise training. In addition, differences in control group settings (eg, basic outpatient rehabilitation in China vs passive follow-ups in the United Kingdom) further contributed to result dispersion.

##### EQ-5D Scores of QoL

The high heterogeneity in EQ-5D scores (*I*^2^=92%) likely stems from a mismatch between general health assessments (mobility and self-care) and COPD-specific interventions (lung function and symptom control). The study by Stamenova et al [24] showed that remote monitoring reduced hospitalizations but had minimal impact on daily activities, while the study by Nyberg et al [27] found increased physical activity improved overall health, though comorbidities diluted these effects. Small sample sizes and varying intervention durations (12 vs 24 months) further exacerbated variability.

##### Overall QoL Assessments

The high heterogeneity in overall QoL assessments (*I*^2^=80%) arises from combining different scales (SGRQ, EQ-5D, and CAT), which vary in structure and sensitivity. For example, the CAT may show improvements in symptoms, while EQ-5D indicates no change or even deterioration. In addition, intervention goals often focus on symptom control or reducing exacerbations (eg, “exacerbation-free weeks” by Boer et al [23]) rather than directly enhancing overall QoL.

##### Self-Efficacy at 12 Months

The high heterogeneity in self-efficacy at 12 months (*I*^2^=91%) observed in the study by Wang et al [21] and the study by Zanaboni et al 2023 [17] primarily stems from key differences in their intervention approaches. The study by Wang et al [21] used a mobile app focusing on education and social support but lacked structured behavioral training, whereas the study by Zanaboni et al 2023 [17] implemented home-based telerehabilitation, incorporating exercise training and real-time monitoring. Differences in assessment timing, adherence strategies, and cultural contexts also contributed to outcome variability, leading to significant heterogeneity in pooled analysis.

##### 6MWT at 3 Months

The high heterogeneity in the 6MWT at 3 months (*I*^2^=94%) is primarily due to fundamental differences in interventions and population adaptability across studies. The study by Robinson et al [34] relied on fully self-guided web-based exercise with community feedback; the study by Tabak et al [29] integrated real-time monitoring with remote counseling; and the study by Wan et al [33] focused on a pedometer combined with an educational website. Furthermore, significant variations in participant age, baseline disease severity, and intervention support intensity collectively led to highly dispersed improvements in walking capacity.

### Relationship With Previous Published Literature

Previous systematic reviews have evaluated the effectiveness of digital health interventions in various areas such as blood pressure control in nondialysis chronic kidney disease [42], prevention of cardiovascular disease [43], hypertension management [44], and weight management in children and adolescents [45]. Many of these reviews concluded that digital health interventions are effective tools that lead to positive outcomes. To our knowledge, 5 other systematic reviews have been published on this topic specifically for patients with COPD [13,46-49]. One of these reviews was limited to the effects of digital health interventions for pulmonary rehabilitation in people with COPD [46], another focused on the role of digital health interventions for the self-management of COPD [47], and the other review focused on the cost-effectiveness of digital health interventions for asthma or COPD [48]. In addition, although 2 recent systematic reviews and meta-analyses aimed to assess the effects of home remote monitoring with mobile apps in patients with COPD [13,49], they included interventions module focusing solely remote monitoring rather than other module (eg, health education, behavioral incentive, and social support), and the technologies do not involve electronic health records, wearable technology, and data analysis platforms. Therefore, this systematic review is unique because it extends beyond previous reviews by including evidence from newly published studies that provide more comprehensive health management services.

### Limitations

This study has several limitations that warrant consideration. First, there may be language biases as the searches were conducted in English, which could restrict the cross-cultural applicability of our findings. Second, our exclusive focus on RCTs—while methodologically rigorous—excluded potentially relevant nonrandomized studies (eg, pragmatic trials) that could provide complementary insights into real-world implementation of digital health tools. Third, the Scopus database was not retrieved, and it should be included in the future. So, we searched PubMed, Embase, Cochrane, and Web of Science databases. Keyword searches in PubMed provide optimal update frequency and include early web-based articles; other databases rank articles by the number of citations as an indicator of importance. In addition, due to the small number of studies, subgroup analyses were not conducted, and publication bias was not explored. Finally, variations in the number of participants, intervention duration, frequency, research tool, and follow-up times led to heterogeneity.

### Practical Implications for Health Care and Further Studies

Digital health interventions show potential as a valuable addition to standard clinical care for COPD. Nevertheless, the adoption of digital health tools in this population is still in its early stages. Further research is essential to evaluate the impact of such interventions. Moreover, it is crucial to determine whether the currently limited effectiveness remains consistent and to explore the conditions under which their benefits could be maximized. These are areas that warrant systematic investigation. In addition, with the growing aging population, future studies should focus on older adults with COPD, who represent the largest group of potential users. Older adults need access to digital health tools to support them to live independently and the self-care of chronic diseases. Computer or smartphone ownership among individuals aged ≥65 years has risen significantly [50]. However, according to the European Union Commission’s 2012 to 2020 eHealth Action Plan, the current mHealth environment lacks intuitive tools and services tailored for older patients [50]. Therefore, gaining insights into the needs of older adults with COPD is critical for designing, developing, and assessing digital health tools interventions for this demographic.

### Conclusions

Our analysis revealed that digital health interventions may have the potential to improve QoL at 3, 6, and 12 months. In addition, the degree of dyspnea significantly decreased at 3 months and approached significance at 12 months. Self-efficacy also improved significantly in the short term, particularly at 3 and 6 months. However, no significant differences were observed in the 6MWT, emergency department admissions, hospital admissions, emergency department admissions for COPD, or hospital admissions for COPD. Furthermore, there is insufficient generalizable evidence to confidently advocate for replacing conventional management strategies with digital health interventions for COPD. This limitation arises due to clinical and methodological variability across studies, small sample sizes, and inconsistencies in platforms functionalities, content, duration, and follow-up protocols. Considering the global surge in digital health tools use and the increasing adoption of eHealth solutions, additional research using robust study designs, long-term follow-ups, and samples representative of older adults is essential to evaluate the long-term sustainability of digital health–based interventions.

## Data Availability

The datasets used and analyzed during this study are available from the corresponding author upon reasonable request.

## Appendix

Multimedia Appendix 1 PRISMA 2020 checklist.

Multimedia Appendix 2 Search strategies.

## Abbreviations

6MWT: 6-minute walk test
AI: artificial intelligence
CAT: Chronic Obstructive Pulmonary Disease Assessment Test
COPD: chronic obstructive pulmonary disease
GSE: General Self-Efficacy
HCP: health care professional
MD: mean difference
MeSH: Medical Subject Headings
mMRC: Modified Medical Research Council
PRISMA: Preferred Reporting Items for Systematic Reviews and Meta-Analyses
QoL: quality of life
RCT: randomized controlled trial
SGRQ: St George’s Respiratory Questionnaire
SMD: standardized mean difference
VAS: visual analogue scale
