# Supplementary material for: Effectiveness of Digital Health Interventions for Chronic Obstructive Pulmonary Disease: Systematic Review and Meta-Analysis
Source: J Med Internet Res. 2025 May 26;27:e76323. doi: 10.2196/76323 (PMC12149779; doi:10.2196/76323)
Supplement: Multimedia Appendix 2 [file jmir_v27i1e76323_app2.doc]

**Online Resource 1.** Search strategies

Search Updated: January 26th, 2023

**PubMed**

| **Search** | **Search Terms/Strategy** | **Results** |
| --- | --- | --- |
| #1 | (((((((Pulmonary Disease, Chronic Obstructive[MeSH Terms]) OR (chronic obstructive pulmonary disease[Title/Abstract])) OR (chronic obstructive lung disease[Title/Abstract])) OR (chronic obstructive airway disease[Title/Abstract])) OR (chronic airflow obstructio[Title/Abstract])) OR (Emphysema[Title/Abstract])) OR (COPD[Title/Abstract])) OR (COAD[Title/Abstract]) | 127306 |
| #2 | (((((((((((((((((((telemedicine[MeSH Terms]) OR (Tele[Title/Abstract])) OR (Mobile Health[Title/Abstract])) OR (mHealth[Title/Abstract])) OR (eHealth[Title/Abstract])) OR (biosensor[Title/Abstract])) OR (remote monitoring[Title/Abstract])) OR (Smartphone[MeSH  Terms])) OR (Smartphone[Title/Abstract])) OR (Phone[Title/Abstract])) OR (Mobile Applications[Title/Abstract])) OR (Mobile Application[Title/Abstract])) OR (App[Title/Abstract])) OR (Internet[MeSH Terms])) OR (Internet[Title/Abstract])) OR (Web[Title/Abstract])) OR (website[Title/Abstract])) OR (digital health[Title/Abstract])) OR (online[Title/Abstract])) OR (information technology[Title/Abstract]) | 795626 |
| #3 | (((((self-management[MeSH Terms]) OR (self care[Title/Abstract])) OR (self management[Title/Abstract])) OR (self monitoring[Title/Abstract])) OR (self-monitoring[Title/Abstract])) OR (self-care[Title/Abstract]) | 65475 |
| #4 | (((random* controlled trial[Publication Type]) OR (controlled clinical trial[Publication Type])) OR (random[Title/Abstract])) OR (trial[Title/Abstract]) | 1626246 |
| #5 | #1 AND #2 AND #3 AND #4 | 144 |

**Embase**

| **Search** | **Search Terms/Strategy** | **Results** |
| --- | --- | --- |
| #1 | 'chronic obstructive lung disease'/exp | 197220 |
| #2 | 'chronic obstructive pulmonary disease':ab,ti OR 'chronic obstructive lung disease':ab,ti OR 'chronic obstructive airway disease':ab,ti OR 'chronic airflow obstructio':ab,ti OR emphysema:ab,ti OR copd:ab,ti OR coad:ab,ti | 186961 |
| #3 | #1 OR #2 | 255040 |
| #4 | 'Telemedicine'/exp | 81878 |
| #5 | tele:ab,ti OR 'mobile health':ab,ti OR mhealth:ab,ti OR ehealth:ab,ti OR biosensor:ab,ti OR 'remote monitoring':ab,ti | 71021 |
| #6 | #4 OR #5 | 141256 |
| #7 | 'smartphone'/exp | 32680 |
| #8 | smartphone:ab,ti OR phone:ab,ti | 86799 |
| #9 | #7 OR #8 | 97292 |
| #10 | 'mobile application'/exp | 31214 |
| #11 | 'mobile application':ab,ti OR app:ab,ti | 67245 |
| #12 | #10 OR #11 | 82806 |
| #13 | 'internet'/exp | 137702 |
| #14 | internet:ab,ti OR web:ab,ti OR website:ab,ti OR 'digital health':ab,ti OR online:ab,ti OR 'information technology':ab,ti | 744003 |
| #15 | #13 OR #14 | 784987 |
| #16 | #6 OR #9 OR #12 OR #15 | 1024690 |
| #17 | 'self care'/exp | 112889 |
| #18 | 'self management':ab,ti OR 'self monitoring':ab,ti OR 'self care':ab,ti | 86408 |
| #19 | #17 OR #18 | 143879 |
| #20 | 'randomized controlled trial'/exp | 864727 |
| #21 | 'random* controlled trial':ab,ti OR 'controlled clinical trial':ab,ti OR random:ab,ti OR trial:ab,ti | 1689737 |
| #22 | #20 OR #21 | 2093862 |
| #23 | #3 AND #16 AND #19 AND #22 | 217 |

**Cochrane Library (CENTRAL)**

| **Search** | **Search Terms/Strategy** | **Results** |
| --- | --- | --- |
| #1 | MeSH descriptor: [Pulmonary Disease, Chronic Obstructive] explode all trees | 8445 |
| #2 | (Pulmonary Disease, Chronic Obstructive):ti,ab,kw OR (chronic obstructive pulmonary disease):ti,ab,kw OR (chronic obstructive lung disease):ti,ab,kw OR (chronic obstructive airway disease):ti,ab,kw OR (chronic airflow obstructio):ti,ab,kw | 20456 |
| #3 | (Emphysema):ti,ab,kw OR (COPD):ti,ab,kw OR (COAD):ti,ab,kw | 21658 |
| #4 | #1 OR #2 OR #3 | 27099 |
| #5 | MeSH descriptor: [Telemedicine] explode all trees | 5219 |
| #6 | (telemedicine):ti,ab,kw OR (Tele):ti,ab,kw OR (Mobile Health):ti,ab,kw OR (mHealth):ti,ab,kw AND (eHealth):ti,ab,kw | 18159 |
| #7 | (biosensor):ti,ab,kw OR (remote monitoring):ti,ab,kw | 2707 |
| #8 | #5 OR #6 OR #7 | 20756 |
| #9 | MeSH descriptor: [Smartphone] explode all trees | 1259 |
| #10 | (Smartphone):ti,ab,kw OR (Phone):ti,ab,kw OR (Mobile Applications):ti,ab,kw OR (Mobile Application):ti,ab,kw OR (App):ti,ab,kw | 37314 |
| #11 | #9 OR #10 | 37314 |
| #12 | MeSH descriptor: [Internet] explode all trees | 6840` |
| #13 | (Internet):ti,ab,kw OR (Web):ti,ab,kw OR (website):ti,ab,kw OR (digital health):ti,ab,kw OR (online):ti,ab,kw | 61299 |
| #14 | (information technology):ti,ab,kw | 5495 |
| #15 | #12 OR #13 OR #14 | 64913 |
| #16 | #8 OR #11 OR #15 | 99661 |
| #17 | MeSH descriptor: [Self-Management] explode all trees | 1367 |
| #18 | (self-management):ti,ab,kw OR (self care):ti,ab,kw OR (self management):ti,ab,kw OR (self monitoring):ti,ab,kw OR (self-monitoring):ti,ab,kw | 72150 |
| #19 | (self-care):ti,ab,kw | 16444 |
| #20 | #17 OR #18 OR #19 | 72150 |
| #21 | MeSH descriptor: [Randomized Controlled Trial] explode all trees | 37 |
| #22 | (random* controlled trial):ti,ab,kw OR (controlled clinical trial):ti,ab,kw OR (random):ti,ab,kw OR (trial):ti,ab,kw | 1188638 |
| #23 | #21 OR #22 | 1188638 |
| #24 | #4 AND #16 AND #20 AND #23 | 329 |

**Web of Science**

| **Search** | **Search Terms/Strategy** | **Results** |
| --- | --- | --- |
| #1 | TS= (Pulmonary Disease, Chronic Obstructive OR chronic obstructive pulmonary disease OR chronic obstructive lung disease OR chronic obstructive airway disease OR chronic airflow obstructio OR Emphysema OR COPD OR COAD) | 206204 |
| #2 | TS= (Telemedicine OR Tele* OR Mobile Health OR mHealth OR eHealth OR biosensor OR remote monitoring OR Internet OR Smartphone* OR Phone* OR Mobile Application* OR App OR Web OR website* OR digital health OR online OR information technology) | 3597965 |
| #3 | TS= (self-management OR self care OR self management OR self monitoring OR self-monitoring OR self-care) | 672005 |
| #4 | TS= (randomized controlled trial OR random* controlled trial OR controlled clinical trial* OR random* OR trial*) | 4763821 |
| #5 | #1 AND #2 AND #3 AND #4 | 516 |
